# Delayed Colonic Perforation Following Blunt Abdominal Trauma: A Diagnostic Dilemma

**DOI:** 10.7759/cureus.111695

**Published:** 2026-06-28

**Authors:** Yash Agrawal, Sanjeev R Chowksey, Brajesh Gupta, Gopal Sarve

**Affiliations:** 1 General Surgery, NKP Salve Institute of Medical Sciences and Research Centre, Nagpur, IND; 2 General Surgery, Government Medical College and Hospital, Nagpur, Nagpur, IND; 3 General Surgery, District General Hospital, Bhandara, Bhandara, IND

**Keywords:** blunt injury abdomen, delayed presentation of perforation, gastrointestinal perforation, sigmoid colon perforation, ­trauma

## Abstract

Hollow viscus injuries following blunt abdominal trauma are relatively uncommon and may present with delayed and masked clinical features, resulting in a delay in diagnosis. A 35-year-old male presented to the hospital after a road traffic accident with isolated blunt abdominal trauma. He was hemodynamically stable with mild abdominal tenderness. Initial ultrasonography and non-contrast computed tomography showed minimal hemoperitoneum without definitive bowel injury or solid organ injury. The patient remained clinically stable for 48 hours except for persistent ileus. A repeat ultrasonography and erect abdominal radiograph subsequently revealed pneumoperitoneum suggestive of a hollow viscus injury. Emergency exploratory laparotomy demonstrated fecal peritonitis due to a traumatic perforation of the descending colon. The perforated damaged colon was resected with resultant end colostomy of the proximal end and blind closure of the distal end. The postoperative course was uneventful. This case highlights the importance of a high index of suspicion for delayed colonic perforation following blunt abdominal trauma. Serial clinical evaluation and repeat imaging are crucial for timely diagnosis and prevention of morbidity.

## Introduction

Blunt abdominal trauma is a common presentation in emergency surgical practice, with solid organ injuries occurring more frequently than hollow viscus injuries [1]. Colonic injuries following blunt trauma are relatively rare, accounting for fewer than 1-5% of abdominal injuries, and are associated with significant morbidity when diagnosis is delayed [1]. Clinical presentation may be masked, particularly in hemodynamically stable patients, and initial radiological investigations may be inconclusive [2]. Delayed perforation of the colon represents a diagnostic challenge, emphasizing the need for vigilant clinical monitoring and repeat imaging [3].

We report a case of delayed traumatic perforation of the descending colon in a hemodynamically stable patient following a road traffic accident, initially managed conservatively, who later developed radiological evidence of pneumoperitoneum necessitating emergency surgical intervention.

## Case presentation

A 35-year-old male presented to the casualty department of the hospital at 7:30 AM on Day 1 with a history of blunt abdominal trauma following a road traffic accident, with the patient being unrestrained by a seatbelt at the time of the accident. There was no history of head injury, loss of consciousness, vomiting, seizures, convulsions, ear-nose-throat bleed, alcohol consumption, or trauma to other body parts.

On presentation, the patient was hemodynamically stable. General and systemic examinations were within normal limits. On examination, pulse rate was 92 beats per minute taken on the right radial artery, and blood pressure was 110/70 mmHg taken on the right brachial artery. Abdominal examination revealed a soft abdomen with mild tenderness in the left hypochondriac region and hypogastrium, without guarding or rigidity. Bowel sounds were present in all quadrants. The patient was admitted under General Surgery and kept under observation. Urethral catheterization revealed a good amount of clear output from the Foley catheter.

Initial blood Investigations revealed no leukocytosis or acute kidney failure. The total leukocyte count was 8,460/mm³ with a normal differential. Serum creatinine was 0.8 mg/dL, blood urea level was 36.7 mg/dL, and all other investigations were within normal limits.

Initial ultrasonography (USG) of the abdomen revealed mild free fluid with internal echoes, suggestive of hemoperitoneum. A non-contrast computed tomography (CT) scan of the abdomen demonstrated an ill-defined collection in the presacral region and lower peritoneal cavity, likely representing hemoperitoneum, with a suspicious fracture of the lower part of the sacrum. Baseline laboratory investigations were within normal limits.

The patient was managed conservatively with intravenous antibiotics, antacids, antiemetics, and analgesics. He remained asymptomatic for the next 48 hours, except for complaints of failure to pass flatus and stools.

A follow-up contrast-enhanced CT of the abdomen and pelvis was performed after 24 hours on day two, which was suggestive of minimal free fluid in the peritoneal cavity, a prominent appendix measuring 10 mm, skin thickening with increased subcutaneous attenuation in the left lumbar region, and bilateral lower lobe airspace opacities suggestive of aspiration pneumonia. This scan showed no other bowel wall abnormalities.

**Figure 1:**
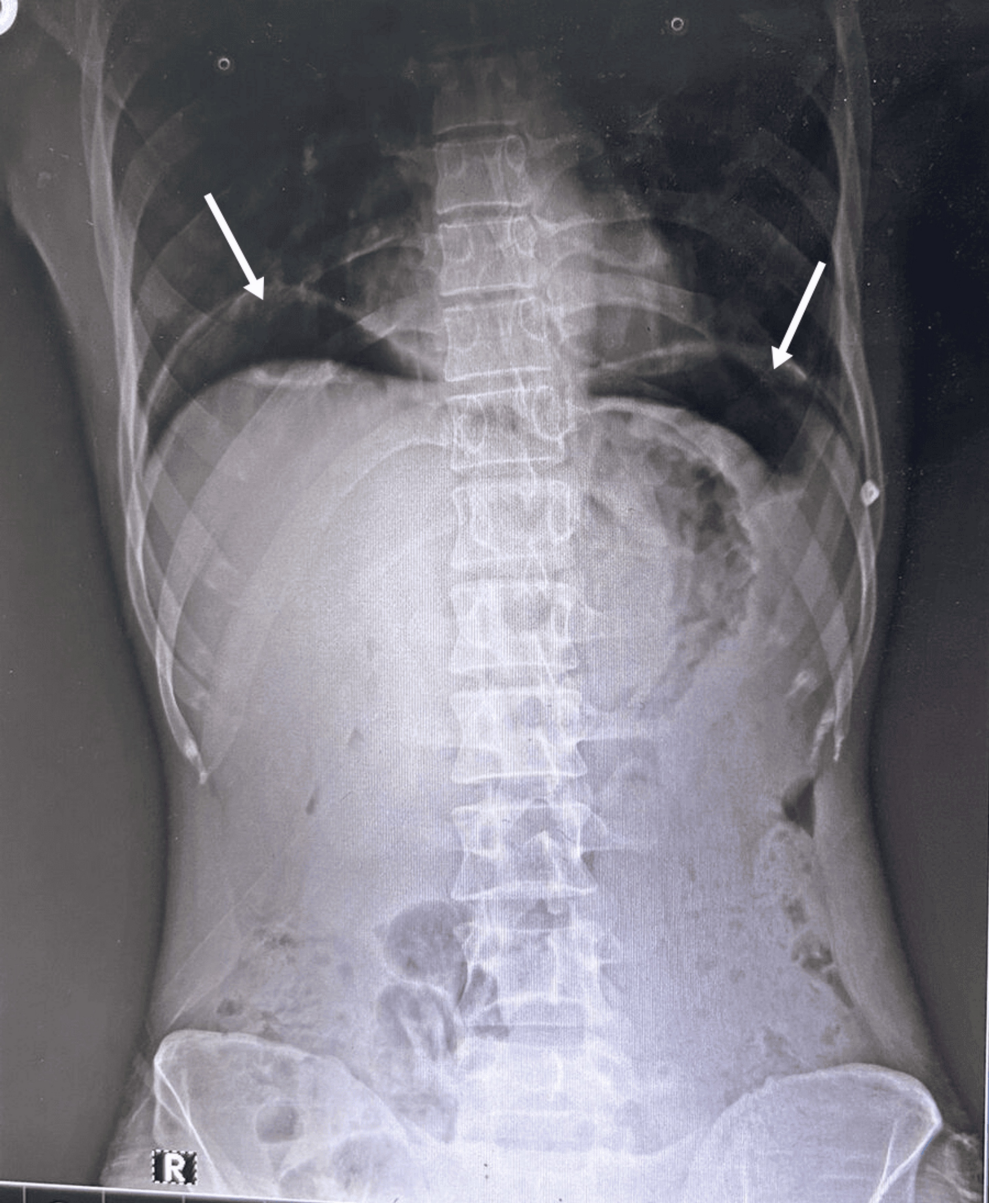
Pneumoperitoneum on abdominal X-ray (erect) performed 72 hours after the admission. White arrows show air under both domes of the diaphragm.

However, a repeat USG performed after 72 hours on day three demonstrated reverberation artifacts anterior to the liver surface, suggestive of pneumoperitoneum, along with mild hemoperitoneum and air foci in the lower abdomen and pelvis. An erect abdominal radiograph confirmed the presence of free air under both domes of the diaphragm (Figure 1).

**Figure 2:**
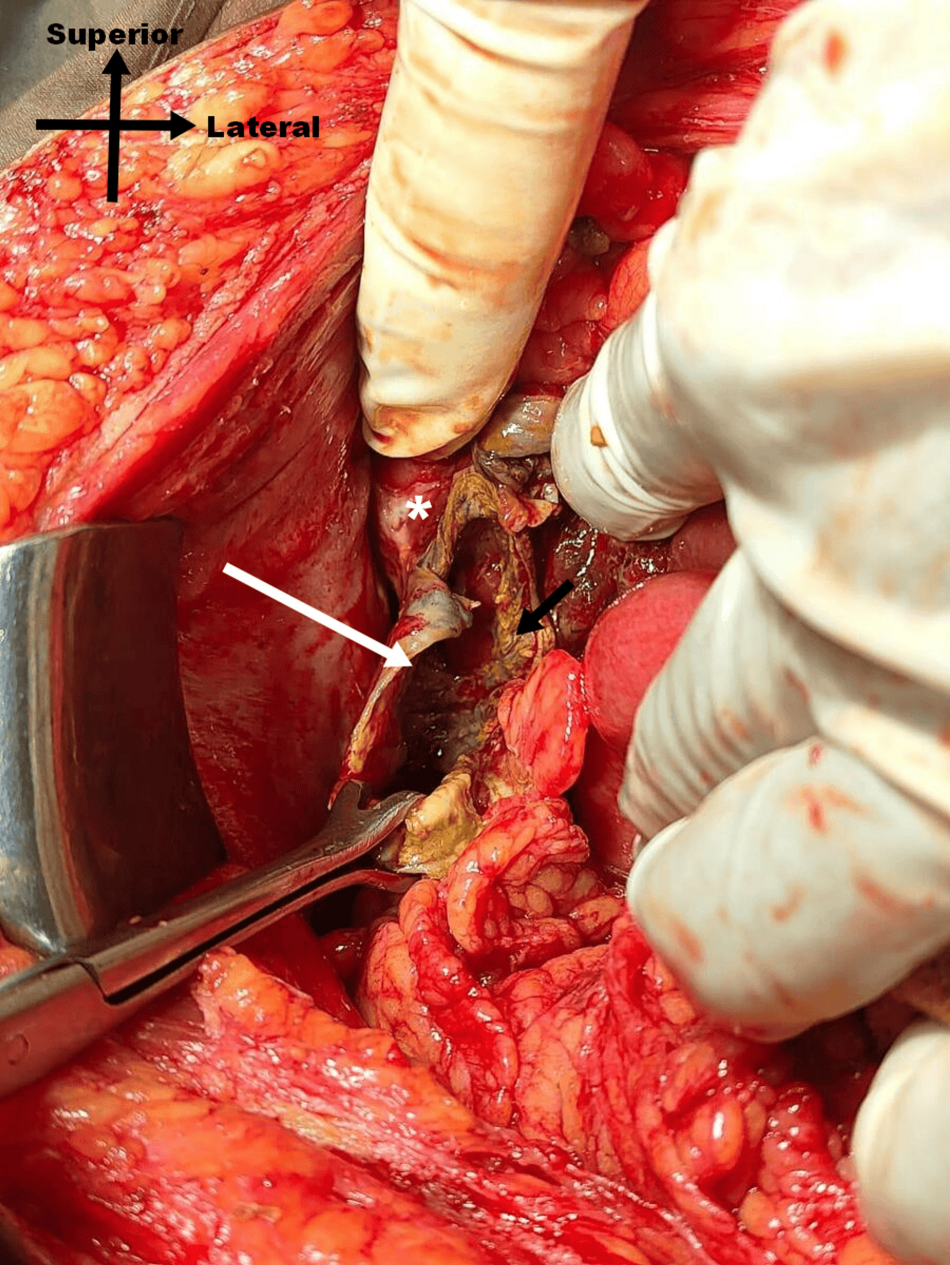
Intraoperative image of the perforation. The white arrow indicates the peroration of the descending colon. The asterisk indicates the proximal end of the bowel. The black arrow indicates the necrotic edematous and friable edges of the perforation.

In view of these findings, the patient was taken to the operating room for an emergency exploratory laparotomy. Intraoperatively, fecal contamination with blood was noted in the peritoneal cavity. A traumatic perforation was identified in the descending colon, approximately 5 cm proximal to the sigmoid colon (Figure 2). There was no evidence of mesenteric tear or vascular compromise. The perforation edges were refreshed, the distal stump was closed, and a proximal end colostomy was fashioned. A thorough warm saline peritoneal lavage was performed, and an abdominal drain (No. 32) was placed.

The postoperative period was uneventful. The colostomy was functional, and the drain was removed subsequently. The patient was discharged in a stable condition, and subsequently continuity was established after three months via a colostomy reversal.

## Discussion

Colonic injuries following blunt abdominal trauma are uncommon and often present a diagnostic dilemma due to delayed or non-specific clinical manifestations [1]. The descending colon is particularly susceptible to deceleration and compression forces against the posterior abdominal wall [4]. Delayed perforation may occur secondary to progressive ischemia, contusion, or transmural necrosis of the bowel wall [4].

Clinical examination alone may be insufficient in detecting early hollow viscus injury, especially in hemodynamically stable patients [3]. Initial imaging studies, including USG and CT, may fail to demonstrate definitive signs of perforation [2]. Although CT is considered the investigation of choice in blunt abdominal trauma, its sensitivity for detecting hollow viscus injury ranges from 55% to 85%, often relying on subtle findings such as minimal free fluid [2,3]. In cases involving the descending colon, the semisolid fecal content may delay leakage of gas and bowel contents, contributing to delayed clinical and radiological presentation [4].

This case emphasizes the importance of serial clinical examinations and repeat imaging in patients with persistent gastrointestinal symptoms such as ileus, even in the absence of overt peritoneal signs, but with persistent ileus [3]. Repeated periodical clinical examination and radiological evaluation can often lead to the detection of colonic perforation. The delayed appearance of pneumoperitoneum on follow-up imaging with appropriate urgent surgical intervention in a timely fashion can prevent the septic complications and associated morbidity and mortality [5].

Surgical management depends on the site of injury, extent of contamination, and hemodynamic status of the patient [4]. In the present case, the presence of fecal contamination and delayed presentation with friable edematous colonic ends warranted diversion with end colostomy, which remains a safe and effective approach in such scenarios [4,5].

Learning points

Hollow viscus injuries, especially colonic perforation, may present late after blunt abdominal trauma despite initial clinical stability. Therefore, normal early imaging, particularly in non-contrasted studies, may not exclude bowel injury. If the patient presents with persistent ileus or masked symptoms, repeat imaging and reassessment are warranted. Therefore, timely surgical intervention and periodic clinico-radiological assessment are crucial to arrive at a diagnosis and the management of such cases.

## Conclusions

Delayed diagnosis of left-sided colonic perforation following blunt abdominal trauma is rare but potentially life-threatening. Even in hemodynamically stable patients with equivocal findings on imaging investigations, the possibility of a hollow viscus injury cannot be ruled out. A high index of suspicion, vigilant clinical monitoring, and timely repeat imaging are essential for diagnosis. Prompt surgical intervention can significantly reduce morbidity and improve outcomes.
